# Effects of submerged liquid fermentation of *Bacillus subtilis* WX‐17 using okara as sole nutrient source on the composition of a potential probiotic beverage

**DOI:** 10.1002/fsn3.1541

**Published:** 2020-06-10

**Authors:** Wai Kit Mok, Yong Xing Tan, Xiao Mei Lyu, Wei Ning Chen

**Affiliations:** ^1^ School of Chemical and Biomedical Engineering Nanyang Technological University Singapore Singapore; ^2^ Interdisciplinary Graduate School Nanyang Technological University Singapore Singapore; ^3^ Advanced Environmental Biotechnology Centre Nanyang Environment and Water Research Institute Nanyang Technological University Singapore Singapore

**Keywords:** antimicrobial activity, antioxidant activity, *Bacillus subtilis*, Metabolomics, okara, probiotic beverage, submerged liquid fermentation, total phenolic content

## Abstract

This work aims to produce a functional probiotic beverage using okara as the sole nutrient source. Hence, okara was fermented with *Bacillus subtilis* WX‐17 in submerged liquid fermentation and the supernatant was tested. Metabolomic analysis showed that the nutritional profile of the beverage was enhanced after fermentation. Essential amino acids as well as short‐chain fatty acids were significantly (*p* < .05) upregulated. Total phenolic content and antioxidant content (in terms of DPPH radical scavenging activity) increased by 6.32 and 1.55 times, respectively. After 6 weeks, probiotic viability remains unchanged when stored at 4°C and the cell count is above the minimum dosage to confer health benefits. Antimicrobial activity was also detected against gram‐positive bacteria. The findings of this work showed the potential of submerged liquid fermentation of *Bacillus subtilis* WX‐17 using okara as sole substrate to produce a functional and low‐cost probiotic beverage.

## Graphical abstract

Submerged liquid fermentation of Bacillus subtilis WX‐17 using okara as sole nutrient source successfully produced a novel probiotic beverage with enhanced nutritional values. Micronutrients, phenolic content, and antioxidant activity were enhanced.

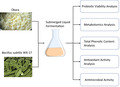

## 1 INTRODUCTION

Okara is the by‐product left behind after the processing of soybean typically in the soymilk and bean curd industry. Okara is generally disposed due to their unpalatable and fibrous nature and is also prone to spoilage due to its high moisture content. However, it is still highly nutritious (okara contains approximately, 50% fiber, 25% of proteins, 10% of lipids as well as a plethora of other useful compounds such as isoflavones, phytates, lignins, saponins, coumestans as well as phytosterols) and studies have found that it is a source of antioxidants and can be potentially used as weight‐loss dietary supplements. Every year, large amount of okara are disposed worldwide in incineration plants and landfills with more than 2.8 million tonnes produced in China's tofu industry alone (Li, Guo, Gao, Wang, & Sun, 2020). It is estimated that almost 14 million tonnes of okara are produced around the world annually (Mok, Tan, Lee, Kim, & Chen, 2019).

In human nutrition, probiotics are defined as live microorganisms in food ingredients that have specific health benefits when consumed in adequate amount (Afzaal et al., 2020). For probiotics to perform, they must remain viable and various researches have shown that okara can support the growth of these microorganisms in model media (Albuquerque, Bedani, Vieira, LeBlanc, & Saad, 2016; Espinosa & Rupérez, 2009; Tu et al., 2014; Vieira, Bedani, Albuquerque, Biscola, & Saad, 2017) as well as under in vitro conditions (Bedani, Rossi, & Isay Saad, 2013). The consumption of fermented soy‐okara products has been credited with numerous health benefits such as the increase in probiotics in the gut microbiota (Cheng et al., 2005) as well as lowering the liver weight, plasma cholesterol levels and hepatic triglyceride content in rats (Kitawaki et al., 2009).

In recent years, researchers are increasingly looking into producing novel probiotic beverages that confer more health benefits to consumers. For instance, (Lu, Tan, Chen, & Liu, 2018) explored the use of *Lactobacillus helveticus* L10*, Lactobacillus paracasei* L26, and *Lactobacillus rhamnosus* HN001 to ferment star fruit juice and found that cell count were around 10^8^ CFU/ml. On top of that, ketones, alcohols, and fatty acids were enhanced which can confer various health benefits. In another study, quinoa, which are traditionally used in salads and stews were processed and fermented separately with *Lactobacillus plantarum* Q823, *Lactobacillus casei* Q11 and *Lactococcus lactis* ARH74. The resultant probiotic beverage (Pasankalla quinoa) was found to contain higher protein content and lower saponin concentration (Ludena Urquizo et al., 2017). Mukisa, Byaruhanga, Muyanja, Langsrud, and Narvhus (2017) utilized single and mixed starter cultures of lactic acid bacteria (LAB) to produce Obushera, a fermented sorghum beverage. It was found that coculturing of LAB with *Saccharomyces cerevisiae* produced a profile flavor compounds that are closed to spontaneous Obushera hence potentially allowing for a more controlled production of Obushera. In the same vein (Men et al., 2019), employed a combination of enzymatic treatment followed by fermentation of jujube juice with *Pediococcus pentasaceus* PC‐5 and *Lactobacillus plantarum* M. It was reported that after fermentation, gamma‐aminobutyric acid as well as branched‐chain and free amino acid levels were enhanced. On top of that, native functional components were also maintained.

The probiotic microorganisms used in fermented beverage studies typically consist of bacteria, such as *Bifidobacterium*, *Lactobacillus,* and *Streptococcus,* the latter two are commonly used in yoghurt. However, these microorganisms typically require additional carbon source such as MRS, the addition of enzymes or other physical processes to break down the cellulose in the food matrix for them to proliferate (Espinosa & Rupérez, 2009; Vieira et al., 2017). This would greatly increase the cost of production of these products on an industrial scale as well as introduce chemicals into the products. As such, there is a gap in terms of the production of a novel probiotic beverage using a low‐cost methodology with minimal addition of chemicals.

*Bacillus subtilis* (*B. subtilis*) is a generally regarded as safe (GRAS) probiotic found in the human gut. It is commonly used in the production of *Natto*, a traditional Japanese fermented food made with soybean whereas rarely explored for the usage as other food products. Previous work has shown that *B. subtilis* is able to utilize okara as the carbon source to grow through solid state fermentation (Ohno, Ano, & Shoda, 1996), Furthermore, *B. subtilis* is known to produce antioxidants and numerous extracellular enzymes such as cellulases, proteases, and lipases which would help to break down cellulose to increase the accessibility of nutrients as well as macromolecules (proteins and lipids) in okara into amino and fatty acids (Lesuisse, Schanck, & Colson, 1993; Mawadza, Hatti‐Kaul, Zvauya, & Mattiasson, 2000; Yang, Shih, Tzeng, & Wang, 2000; Zhu, Fan, Cheng, & Li, 2008). However, to the best of our knowledge, submerged liquid fermentation of *B. subtilis* using okara as sole nutrient source as well as its development for producing a probiotic beverage, has not been evaluated. In this work, submerged liquid fermentation of *Bacillus subtilis* WX‐17 (*B. subtilis* WX‐17) with okara as sole nutrient source is performed to develop a potential low‐cost and functional probiotic beverage. The viability of the probiotic *B. subtilis* WX‐17 as well as the nutritional analysis, total phenolic content, antioxidant, and antimicrobial activity assays of fermented product are investigated herein to verify the practicality of this approach. Moreover, the metabolic mechanism of *B. subtilis* WX‐17 for submerged liquid fermentation of okara is analyzed.

## 2 MATERIALS AND METHODS

### 2.1 Materials

All chemicals including nutrient broth, methanol, ribitol, methoxamine hydrochloride, N‐methyl‐N‐(trimethylsilyl) trifluoroacetamide (MSTFA), trimethylchlorosilane (TMCS), Folin Ciocalteu's reagent, 20% sodium carbonate, 1‐1,‐diphenyl‐2‐picryl‐hydrazil (DPPH), ethanol, 6‐hydroxy‐2,5,7,8‐tetramethylchroman‐2‐carboxylic acid (Trolox), and gallic acid were purchase from Sigma‐Aldrich. Fresh okara samples were kindly provided by Vitasoy International Singapore Pte Ltd, Singapore. Okara were separated into aliquots and sealed in airtight polyethylene bags and stored at −20°C in the dark.

### 2.2 Fermentation

Ten g of heat‐treated okara was placed in a flask and inoculated with *B. subtilis* WX‐17 which was isolated and identified as described previously (Mok et al., 2019) at a concentration of 10^6^ CFU/g of okara. Fifty millilitre of sterile water were added to the flask, and subsequently submerged liquid fermentation was carried out for 72 hr at 37°C and 200 rpm. After fermentation, okara was removed through filtration and the supernatant was stored in 4°C until further analysis. Okara with 50 ml of sterile water and no inoculation of *B. subtilis* WX‐17 were used as controls.

### 2.3 Enumeration of *B. subtilis* WX‐17

Hundred microlitre of sample were added to 900 µl of sterile water and serial diluted 10 times. Hundred microlitre from each dilution were plated onto nutrient agar plates and incubated at 37°C for 24 hr. After which the cell counts were recorded. This is repeated weekly for a duration of 6 weeks.

### 2.4 Metabolomic analysis

Analysis of the metabolites present in the samples was performed based on the method described by Cooray, Lee, and Chen (2017) with minor modifications. Briefly, 10 µl of ribitol were added to 1.5 ml of sample and freeze‐dried. Samples were derivatized using 100 µl of methoxamine hydrochloride and silylation was carried out using 200 µl of *N*‐methyl‐*N*‐(trimethylsilyl) trifluoroacetamide (MSTFA) with 1% trimethylchlorosilane (TMCS). Samples were then centrifuged, and 120 µl were transferred to glass vials for gas chromatography mass spectrometry (GC‐MS) analysis. The column and method used were as per described by Cooray et al. (2017).

### 2.5 Total phenolic content analysis

Total phenolic content analysis was carried out with respect to the protocol described by Kamtekar, Keer, and Patil (2014). One millilitre of sample was added with 5 ml of deionized water, 0.5 ml of Folin Ciocalteu's reagent and shaken. After 5 min, 1.5 ml of 20% sodium carbonate was added and made up to 10 ml before incubation for 2 hr. The absorbance of the mixture was measured at 750 nm with deionized water as blank using Nanodrop 2000c Spectrophotometer.

### 2.6 DPPH scavenging activity analysis

The DPPH radical scavenging activity of the samples was evaluated using the method described by Gjorgievski, Tomovska, Dimitrovska, Makarijoski, and Shariati (2014) with minor modification. Six hundred microlitre of sample were added with equal volume of DPPH solution and vortexed before incubation in the dark for 30 min. Absorbance was measured at 515 nm using ethanol as blank, and the activities of the samples were evaluated with respect to trolox equivalent‐% signal inhibition calibration curve whereby % signal inhibition is defined as: $\%\;\text{Signal}\;\text{Inhibition}=\left(1-\frac{A_{s}}{A_{o}}\right)\times 100$. *A*_s_ is defined as the absorbance of the samples and *A*_o_ is defined as the absorbance of pure DPPH.

### 2.7 Agar disk diffusion for antimicrobial assay

The antimicrobial activity of the fermented okara probiotic beverage (FO) was evaluated by employing the agar disk diffusion assay as documented by Ng, Lyu, Mark, and Chen (2019) with minor modifications. Firstly, *Escherichia coli* (*E. coli*) ATCC 25922 and *Staphylococcus aureus* (*S. aureus*) ATCC 29213 were streaked onto fresh LB agar plates and incubated overnight at 37°C. After incubation, individual colonies from each culture were resuspended in 1 ml of Mueller‐Hinton (MH) broth and the OD600 was adjusted to approximately 0.5 McFarland standard which corresponds to 1–3 × 10^8^ CFU/ml. A sterile cotton swab was then used to streak‐inoculate each strain on fresh MH agar plates. Sterile filter paper disks of 6 mm were inoculated with 5 and 10 µl of FO as well as the unfermented control (RO) and subsequently placed onto the inoculated plates. Thereafter, all inoculated plates were incubated at 37°C for 18 hr. The antimicrobial activity was determined by measuring the inhibition zones in millimeters (mm) on the agar plates less the diameter of the filter paper (6 mm). All disk diffusion assays were carried out in triplicates.

### 2.8 Statistical analysis

Statistical analysis was performed using Metaboanalyst 4.0 as described previously (Mok et al., 2019). Data scaling was carried out using mean‐centering and divided by the standard deviation of each variable prior to partial least‐squares discriminant analysis (PLS‐DA) and heat map analysis. The heat map was constructed using Euclidean distance measurement and ward clustering algorithm. All experiments were carried out in triplicates.

## 3 RESULTS AND DISCUSSION

### 3.1 Viability of *B. subtilis* WX‐17

**FIGURE 1:**
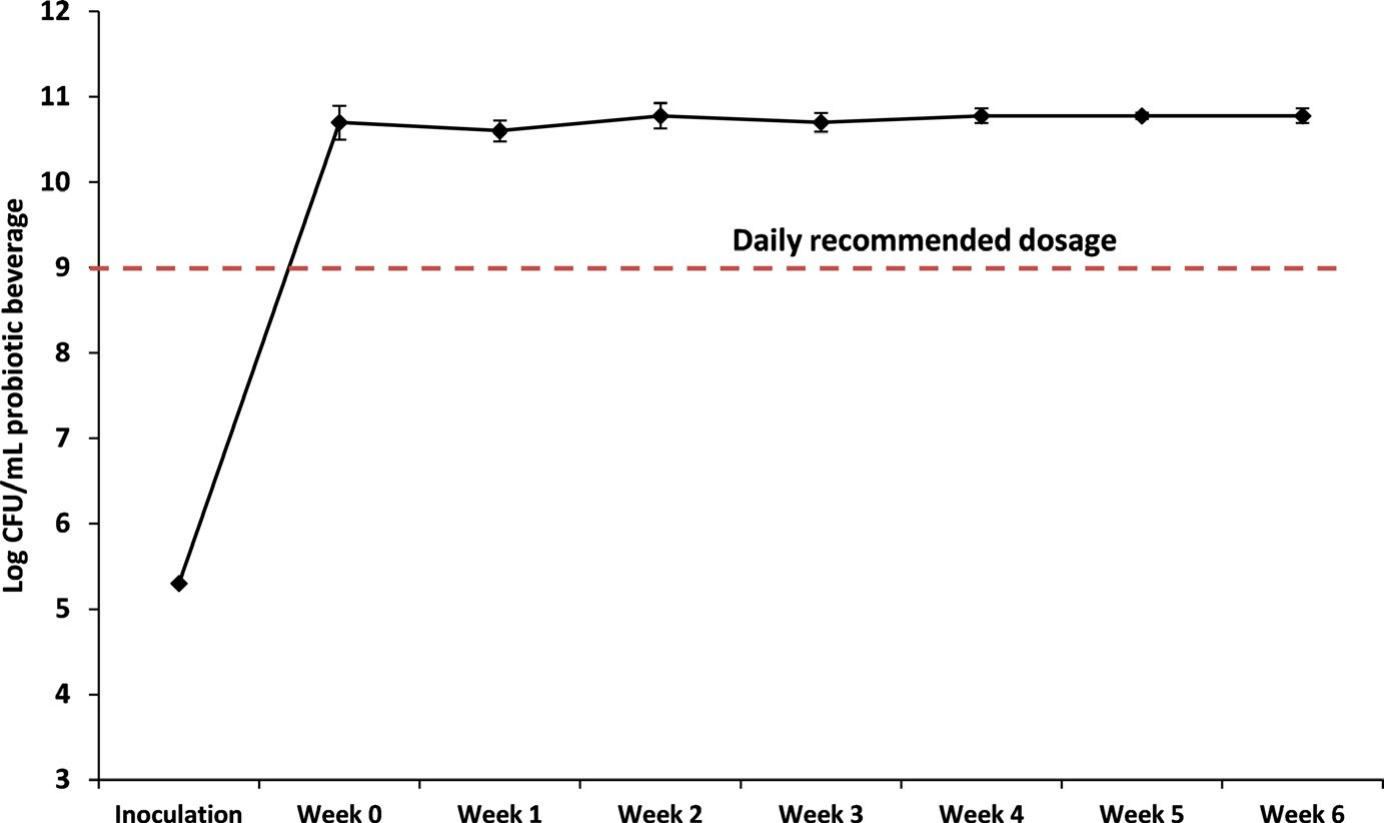
Changes in cell count of *Bacillus subtilis* WX‐17 using okara as sole nutrient source when stored at 4°C for 6 weeks

In a probiotic beverage, it is important that the microorganism remains viable after prolonged period of storage. The viability of the probiotic strain *B. subtilis* WX‐17 was tested for a duration of 6 weeks at storage temperature of 4°C (Figure 1). Results have shown that after 6 weeks of storage the cell count of viable *B. subtilis* WX‐17 remained relatively unchanged at an average of 10.65 log CFU/ml which is above the daily probiotic recommended intake amount of 9 log CFU (Hill et al., 2014). These results are due to the fact that *B. subtilis* WX‐17 would form endospores under unfavorable growth conditions (McKenney, Driks, & Eichenberger, 2012). This property would come in handy in terms of increasing the shelf life of the beverage without the addition of other ingredients which shows the practicality of the methodology in the development of a low‐cost functional *B. subtilis* probiotic beverage.

### 3.2 Metabolic profiling of fermented okara probiotic beverage and unfermented okara control

**FIGURE 2:**
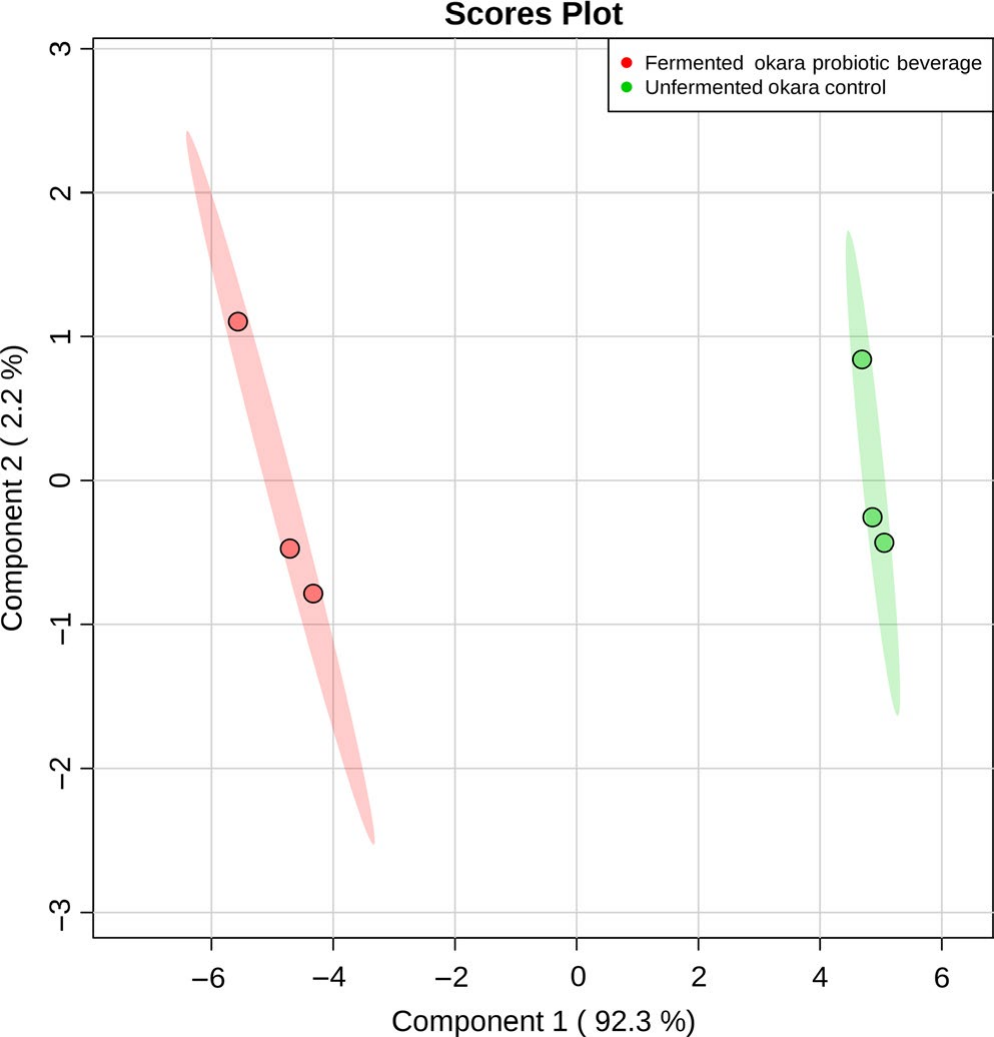
PLS‐DA score plot of all metabolites found for fermented okara probiotic beverage and unfermented okara control. The green and red highlights denoted the 95% confidence region. Explained variance is shown in brackets

A metabolomic analysis was carried out using the GC‐MS between FO and RO to better understand the difference in the metabolic profiles during the fermentation process. Statistical analysis in the form of a PLS‐DA and heat map was conducted to understand these changes better. A total of 31 metabolites were detected. PLS‐DA analysis (Figure 2) was carried out to show the difference in the metabolic profiles. The green and red highlights represent the 95% confidence region. The first principal component accounted for 92.2% of the total variance while the second accounted for 2.2% for a total of 94.4%. This shows that the first principal component largely explained most of the variance within the dataset. The PLS‐DA score plot (*R* ^2^ = 99.9% and *Q* ^2^ = 98.6%) showed a clear and distinct separation between the two sets of samples along the first principal component and not along the second principal component which is acceptable since most of the variance are accounted for by the first component.

**FIGURE 3:**
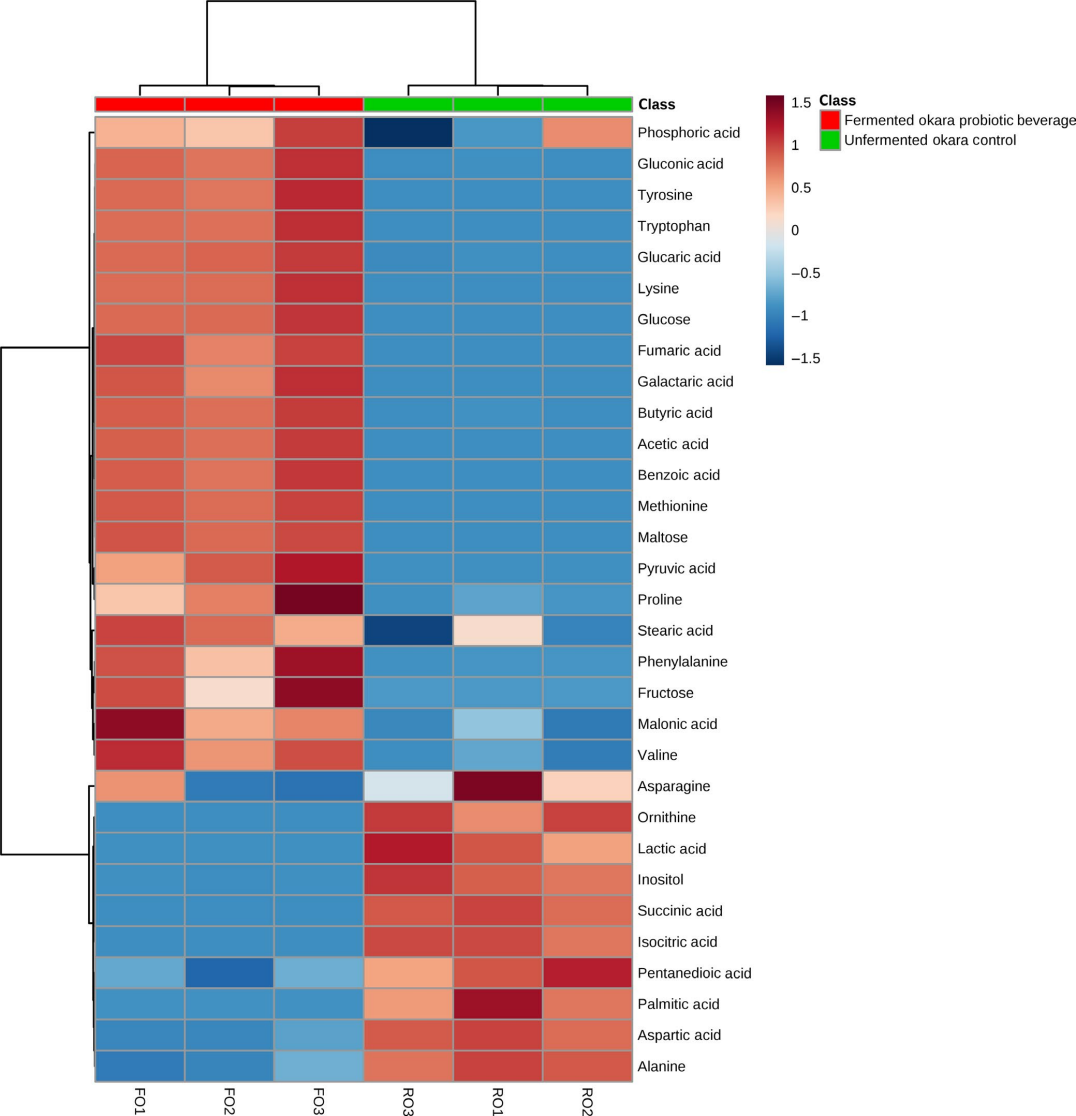
Heatmap analysis correlating the metabolites of fermented okara probiotic beverage and unfermented okara control. Metabolites in brown are upregulated while those in blue are downregulated

A clustering heat map (Figure 3) was constructed to provide a visual representation of the changes in each metabolite between FO and RO. From the heat map, it can be seen that the overall profile changed significantly after submerged liquid fermentation by *B. subtilis* WX‐17 (Figures 2 and 3; Table S1). Firstly, 11 amino acids were detected in the samples of which seven were upregulated (tyrosine, tryptophan, lysine, methionine, proline, phenylalanine, and valine) after fermentation while four were downregulated. Interestingly, the amino acids that were downregulated (alanine, aspartic acids, asparagine, and ornithine) were nonessential amino acids (the amino acids naturally produced by the human body in adequate amount; Eagle, 1959). Since these nonessential amino acids are glucogenic, it can be hypothesized that they were consumed by *B. subtilis* WX‐17 for metabolism. This hypothesis is reinforced by the observation that both glucose, maltose, and fructose were upregulated after fermentation which suggested that the rate of consumption of carbohydrates by *B. subtilis* WX‐17 was lower than the rate of production since the microorganism are well known to produce amylase. This hypothesis is supported in a study by Sheu, Konings, and Freese (1972) which suggested that the presence of short‐chain fatty acids (SCFAs) would inhibit the uptake of carbohydrates. Therefore, it is possible that *B. subtilis* WX‐17 was not able to fully utilize the carbohydrates available for metabolism and hence used the nonessential amino acids instead. Vong and Liu (2019) also related that essential amino acids increased when okara is fermented by *Lindnera saturnus* under submerged liquid condition.

Two important SCFAs in acetic acid and butyric acid were also detected in FO. This is in agreement with studies conducted by Dirar, Harper, and Collins (1985; Haq et al., 2018) which showed that *B. subtilis* can produce SCFAs in the presence of dietary fibers. The upregulation of these SCFAs are important as they are known to confer various health benefits such as reducing the risk of colonic cancer, cardiovascular diseases as well as various gastrointestinal disorders (Hijová & Chmelarova, 2007; Wong, de Souza, Kendall, Emam, & Jenkins, 2006).

On top of that, other important metabolites with numerous purported health benefits were also upregulated after fermentation. For example, both gluconic and glucaric acids are commonly found in *Kombucha* which have been linked with detoxification of toxins from the body (Martínez Leal, Valenzuela Suárez, Jayabalan, Huerta Oros, & Escalante‐Aburto, 2018). Fumaric acid was found to be effective in the treatment of psoriasis (Altmeyer et al., 1994). Studies have also shown that pyruvic acid exhibited angiogenic activity in both in vivo and in vitro models (Lee et al., 2001).

These results suggested that *B. subtilis* WX‐17 were able to utilize okara as the sole nutrient source in submerged liquid fermentation and produce enzymes such as amylases, proteases, and lipases to break down carbohydrates, proteins, and lipids into their simpler form (Asgher, Asad, Rahman, & Legge, 2007; Lesuisse et al., 1993; Yang et al., 2000).

The metabolites were also expressed in terms of relative percentage (Table S1) to gain a better context of the respective metabolic profiles. It is telling that many of the metabolites detected in FO were undetected in RO. The bulk of the metabolites in FO are made up of phenylalanine (17.52%), lysine (25.12%), and glucaric acid (15.10%) while RO consists of mainly lactic acid (57.46%), isocitric acid (18.84%), and myo‐inositol (15.57%)

### 3.3 Total phenolic content and antioxidant activity

**FIGURE 4:**
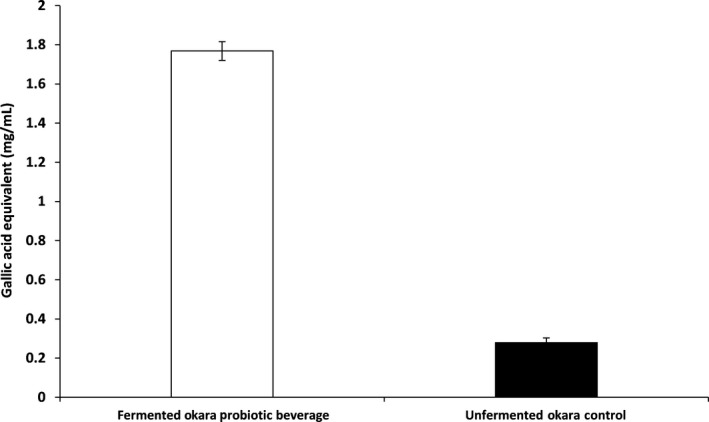
Total phenolic content of fermented okara probiotic beverage and unfermented okara control expressed in terms of gallic acid equivalent (mg/ml). Each datapoint represents the triplicate mean. Error bars represent standard deviation

Total phenolic content of FO (Figure 4) was found to have increased significantly compared to RO. The increase in total phenolic content after fermentation is likely due to the release of enzymes by *B. subtilis* WX‐17 which hydrolyzes the phenolic complexes which are combined or bound with sugars into soluble‐free phenols (Queiroz Santos et al., 2018). FO was found to contain approximately 1.77 mg/ml of phenolic content expressed in terms of gallic acid equivalent while RO contains only 0.28 mg/ml. This marks an increase in total phenolic content of 6.32 times. Similarly, Rashad, Mahmoud, Abdou, and Nooman (2011) reported that okara fermented with various GRAS microorganism showed a significant increase in total phenolic content.

Okara is well known to contain large number of phenolic compounds. Studies have shown that phenolic molecules have numerous health benefits such as antioxidation, antiaging, antiinflammation, anticarcinogenic, and antiatherosclerosis.

**FIGURE 5:**
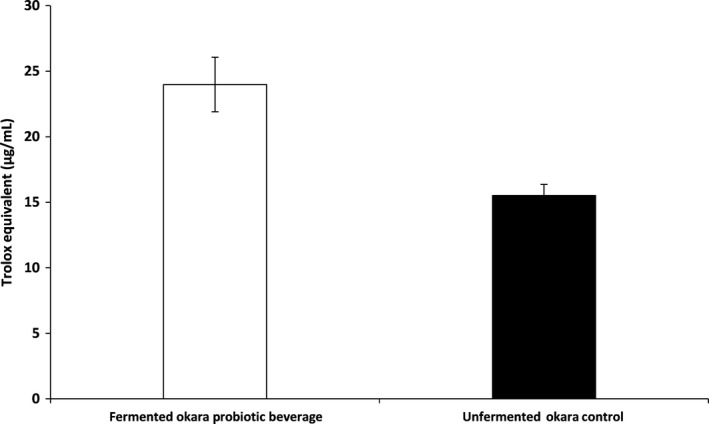
DPPH scavenging activity of fermented okara probiotic beverage and unfermented okara control expressed in terms of Trolox equivalent (µg/ml). Each datapoint represents the triplicate mean. Error bars represent standard deviation

The antioxidant activity of FO (Figure 5) was also evaluated with regard to its DPPH radical scavenging activity. For FO, the DPPH radical scavenging activity was found to be 23.99 µg trolox equivalent/ml while RO contains only 15.50 µg trolox equivalent/mL which translates to an increase of 1.55 times.

Our body generates substantial amount of free radical through metabolic processes which may result in oxidative damages to the tissues and cells in the human body. Therefore, the increase in DPPH radical scavenging activity (Figure 5) by 1.55 times after fermentation by *B. subtilis* WX‐17 is ideal. This observation is in agreement with studies by Juan and Chou (2010) whom reported that soybean fermented with *B. subtilis* B2 exhibited an increase in DPPH radical scavenging activity.

### 3.4 Antimicrobial activity

**FIGURE 6:**
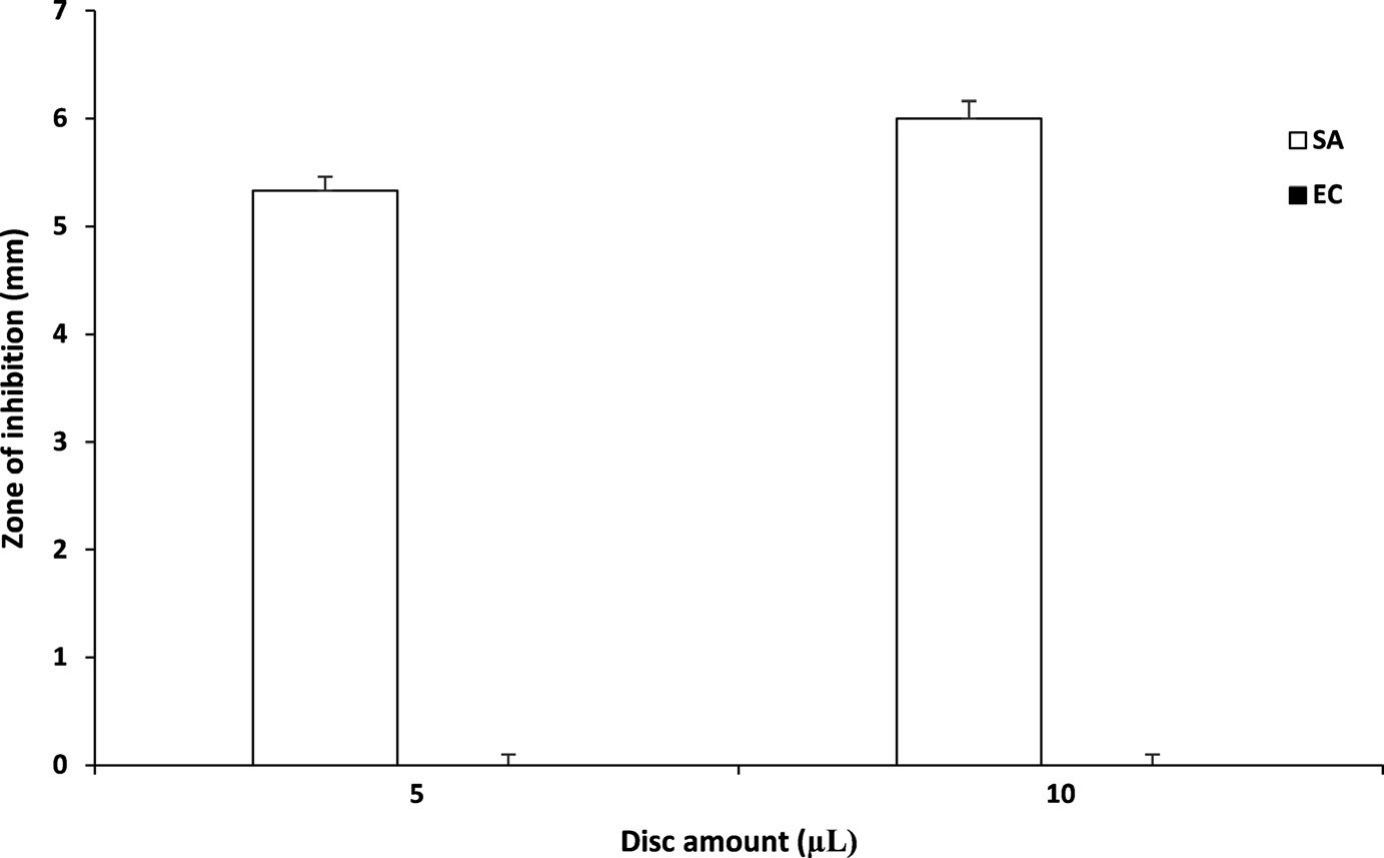
Agar disk diffusion results for *E. coli* ATCC 25922 and *S. aureus* ATCC 29213. Each datapoint represents the triplicate mean. Error bars represent standard deviation. SA: *S. aureus* ATCC 29213. EC: *E. coli* ATCC 25922

FO showed antimicrobial activity against *S. aureus* ATCC 29213 which is a gram‐positive bacterium with mean inhibition zones of 5.3 and 6 mm for 5 and 10 µl respectively (Figure 6). No inhibition zones were detected for *E. coli* ATCC 25922 which indicates that there was no antimicrobial activity. RO did not exhibit any antimicrobial activity for both *E. coli* ATCC 25922 and *S. aureus* ATCC 29213. These results are supported by Ghribi et al. (2012) and Yilmaz, Soran, and Beyatli (2006) which suggested that *B. subtilis* can exhibit antimicrobial properties due to the production of broad‐spectrum antibiotics or biosurfactants such as bacillomycins which are amphiphilic membrane‐active biosurfactants with strong antimicrobial activities. However, Ghribi et al. (2012) also suggested that the type of substrate affects the production of biosurfactants which could explain why the fermented okara probiotic beverage only exhibited antimicrobial activity against gram‐positive microorganisms. These findings suggest that less chemical preservatives might be required for the probiotic beverage from a commercialization point of view which would reduce cost and public perceptions.

## 4 CONCLUSION

This work showed that submerged liquid fermentation with *B. subtilis* WX‐17 using okara as the sole nutrient source is a viable approach. FO exhibited higher amount of amino acids, SCFAs as well as other useful metabolites such as gluconic acid, glucaric acid, and fumaric acids. It also provides an adequate amount of probiotic (more than 9 log CFU daily) on top of increased amount of phenolic content and antioxidant activity. Furthermore, FO displayed antimicrobial activity against gram‐positive bacteria which could potentially reduce the need for chemical preservatives in the probiotic beverage. These were achieved without the addition of other enzymes or chemicals which would greatly reduce the cost of production as well as minimize potential health concerns for our increasingly health conscious population. It would be of significant interest to further investigate the health benefits, consumer acceptance of this fermented okara probiotic beverage as well as modifications to the substrate such that the antimicrobial activity encompasses gram‐negative bacteria.

## CONFLICT OF INTEREST

The authors declare that they have no competing interests.

## AUTHOR’S CONTRIBUTIONS

Chen WN conceived the motivation behind the project. Mok WK and Tan YX designed and performed the experiments as well as analyzed the data. Lyu XM provided technical advice on experimental set‐up and data analysis. Mok WK and Tan YX wrote the manuscript. Lyu XM and Chen WN revised the manuscript. All authors read and approved the final manuscript.

## Supporting information

Table S1
