# Supplementary material for: Effects of submerged liquid fermentation of Bacillus subtilis WX‐17 using okara as sole nutrient source on the composition of a potential probiotic beverage
Source: Food Sci Nutr. 2020 Jun 10;8(7):3119–27. doi: 10.1002/fsn3.1541 (PMC7382164; doi:10.1002/fsn3.1541)
Supplement: Supplementary file 1 — Table S1 [file FSN3-8-3119-s001.docx]

**Table S1.** Changes in metabolites in terms of relative percentage for fermented okara probiotic beverage and unfermented okara control.

| Metabolites  Relative % | Fermented Okara Probiotic Beverage | Unfermented Okara Control |
| --- | --- | --- |
| Lactic Acid ^a^ | undetected | 57.46±2.77 |
| Acetic Acid ^a^ | 2.47±0.07 | undetected |
| Pyruvic Acid ^a^ | 0.16±0.02 | undetected |
| Butyric Acid ^a^ | 2.78±0.01 | 0.18±0.02 |
| Malonic Acid ^a^ | 0.24±0.02 | 0.08±0.01 |
| Valine ^a^ | 0.10±0.01 | 0.03±0.003 |
| Succinic Acid ^a^ | 0.09±0.004 | 1.43±0.13 |
| Fumaric Acid ^a^ | 0.17±0.01 | undetected |
| Methionine ^a^ | 0.50±0.02 | undetected |
| Proline ^a^ | 0.64±0.04 | 0.21±0.02 |
| Aspartic Acid ^a^ | 0.12±0.004 | 0.14±0.01 |
| Alanine ^a^ | 0.31±0.02 | 0.34±0.04 |
| Pentanedioic Acid ^a^ | 1.72±0.09 | 1.19±0.18 |
| Benzoic Acid ^a^ | 3.92±0.09 | undetected |
| Phenylalanine ^a^ | 17.52±2.28 | 1.14±0.17 |
| Lysine ^a^ | 25.12±0.83 | undetected |
| Phosphoric Acid | 0.36±0.01 | 0.15±0.05 |
| Ornithine ^a^ | undetected | 1.20±0.19 |
| Isocitric Acid ^a^ | 0.38±0.01 | 18.84±1.11 |
| Fructose ^a^ | 0.62±0.14 | Undetected |
| Tyrosine ^a^ | 6.40±0.12 | Undetected |
| Galactaric Acid ^a^ | 0.74±0.02 | Undetected |
| Gluconic Acid ^a^ | 5.24±0.11 | 0.27±0.03 |
| Palmitic Acid ^a^ | undetected | 0.39±0.08 |
| Tryptophan ^a^ | 1.37±0.04 | undetected |
| Stearic Acid ^a^ | 0.93±0.10 | 0.35±0.06 |
| Asparagine | 0.24±0.05 | 0.15±0.03 |
| Myo-Inositol ^a^ | 1.57±0.11 | 15.57±0.77 |
| Glucaric Acid ^a^ | 15.10±0.75 | 0.86±0.12 |
| Glucose ^a^ | 7.58±0.26 | undetected |
| Maltose ^a^ | 3.60±0.19 | undetected |

*Results are as mean ± standard deviation (3 replicates)*

*^a^ denotes metabolites that are significantly different (p<0.05)*
